# Nanotechnology and Glycosaminoglycans: Paving the Way Forward for Ovarian Cancer Intervention

**DOI:** 10.3390/ijms19030731

**Published:** 2018-03-04

**Authors:** Yasar Hoosen, Priyamvada Pradeep, Pradeep Kumar, Lisa C. du Toit, Yahya E. Choonara, Viness Pillay

**Affiliations:** Wits Advanced Drug Delivery Platform Research Unit, Department of Pharmacy and Pharmacology, School of Therapeutic Sciences, Faculty of Health Sciences, University of the Witwatersrand, Johannesburg, 7 York Road, Parktown 2193, South Africa; yasar.hoosen@students.wits.ac.za (Y.H.); priyamvada.pradeep@wits.ac.za (P.P.); pradeep.kumar@wits.ac.za (P.K.); lisa.dutoit@wits.ac.za (L.C.d.T); yahya.choonara@wits.ac.za (Y.E.C.)

**Keywords:** ovarian cancer, nanomedicine, glycosaminoglycans, antibodies, chemotherapy

## Abstract

Ovarian cancer (OC) has gained a great deal of attention due to its aggressive proliferative capabilities, high death rates and poor treatment outcomes, rendering the disease the ultimate lethal gynaecological cancer. Nanotechnology provides a promising avenue to combat this malignancy by the niche fabrication of optimally-structured nanomedicines that ensure potent delivery of chemotherapeutics to OC, employing nanocarriers to act as “intelligent” drug delivery vehicles, functionalized with active targeting approaches for precision delivery of chemotherapeutics to overexpressed biomarkers on cancer cells. Recently, much focus has been implemented to optimize these active targeting mechanisms for treatment/diagnostic purposes employing nanocarriers. This two-part article aims to review the latest advances in active target-based OC interventions, where the impact of the newest antibody, aptamer and folate functionalization on OC detection and treatment is discussed in contrast to the limitations of this targeting mechanism. Furthermore, we discuss the latest advances in nanocarrier based drug delivery in OC, highlighting their commercial/clinical viability of these systems beyond the realms of research. Lastly, in the second section of this review, we comprehensively discussed a focus shift in OC targeting from the well-studied OC cells to the vastly neglected extracellular matrix and motivate the potential for glycosaminoglycans (GAGs) as a more focused extracellular molecular target.

## 1. Introduction

Ovarian cancer (OC) is a highly lethal disorder, responsible for more deaths than all other gynaecological cancers collectively with over 220,000 cases recorded worldwide annually [1]. The poor prognosis of OC is attributed to its vicious metastasising ability, resulting in approximately 75% of patients being diagnosed in the advanced FIGO (Federation of Gynecology and Obstetrics) stages, in addition to high rates of relapse and inefficacious treatments [2,3,4]. Despite the current gold standard treatments for OC (carboplatin and paclitaxel) and standard primary debulking surgery (PDS), only 20–25% of cases are cured effectively [5]. Although the majority of women respond well to treatment initially, recurrence, unbearable side effects and drug resistant challenges the management and treatment of OC. 

Nanotechnology is the synthesis of materials and devices that manipulate matter at an incredibly small scale—between 1 and 100 nm [6,7]. Nanotechnology holds great promise for revolutionizing the field of oncology by the development of new drug delivery systems such as several nanocarriers [8] as well their use in diagnosis, imaging, synthetic vaccine development and miniature medicals [9]. 

Nanocarriers include organic and inorganic nanomaterials (gold, iron, silver, cerium, titanium dioxide, and silica), non-degradable and biodegradable polymers as well as lipids, e.g., liposomes, nanoemulsions and solid-lipid nanoparticles (NPs). Self-assembling amphiphilic molecules, dendrimers, metal inorganic semiconductor nanocrystals and carbon nanotubes are also employed as nanocarriers for medicinal and diagnostic purposes [10,11]. Nanocarriers display favourable abilities to encapsulate drug molecules to enhance the delivery of poorly soluble drugs. Furthermore, their higher surface area to volume ratio offers enhanced half-life circulation time and pharmacokinetics, improved tissue distribution, decreased non-specific toxicities and reduced incidence of resistance [12,13,14]. These systems can preferentially deliver drugs to targeted locations, in contrast to systematic administration of free drugs [15]. The combination of these nanocarriers with active targeting approaches for specific molecular targets provides precision delivery of chemotherapeutics. 

This two-part review article aims to discuss the latest advances in molecular target specific nanocarriers employing active targeting strategies including aptamer, antibody and folate (FA) functionalization, as an update to our research group’s previous publications [16,17] for OC treatment and detection in Section 1, Section 2 and Section 3 of the article. Section 4 of the article critically evaluates the potential for glycosaminoglycans (GAGs) as a more focused OC molecular target, where we discuss their clinical roles in OC and the potential interventions to target them.

## 2. Active Targeting in Ovarian Cancer 

**Figure 1:**
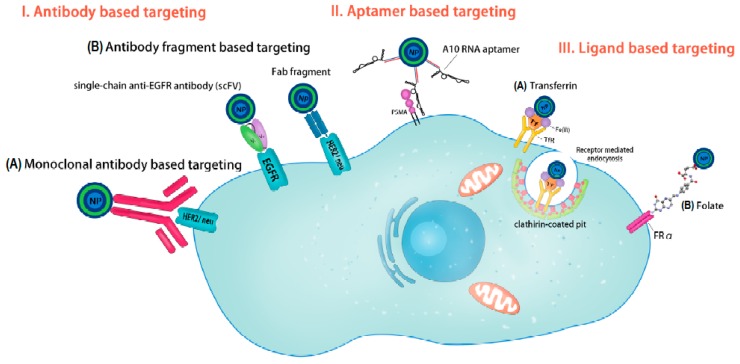
A schematic depicting method employed for active targeting of nanoparticles. I. Antibody-based targeting, which involves the use of: (A) monoclonal antibodies such as anti-Her2/neu antibody directed toward Her2/neu receptors on the target cell membrane; and (B) antibody fragments: single-chain variable fragments (scFV) such as single-chain anti-epidermal growth factor receptor (EGFR) antibody directed toward EGFR, or antigen-binding fragment (Fab) such as anti-Her2/neu Fab. II. Aptamer-based targeting such as the A10 RNA aptamer directed toward prostate-specific membrane antigen (PSMA) on the surface of the target cells. III. Ligand-based targeting such as: (A) transferrin-based targeting of nanoparticles toward transferrin receptors where uptake of the nanoparticles takes place through receptor-mediated endocytosis through clathrin-coated pits; and (B) folate-based targeting using folic acid (FA) to target folate receptor alpha (FRα), which is upregulated on the surface of neoplastic cells. (Reprinted from [23] with permission from Cancer Research Clinical Oncology).

Despite the current advances in chemotherapeutics, the common flaw i.e., the lack of biological specificity leads to failed treatment outcomes [18,19], consequently resulting in uncontrolled bio distribution of chemotherapeutics to healthy tissues [20]. Overexpressed molecular targets are exploited, employing polyvalent engineered nanocarriers encompassing a targeting ligand for enhanced affinity, spatial localization and therapeutic efficacy of chemotherapeutics at the lowest possible dosages [16], following their uptake into the desired cell via receptor mediated endocytosis [21,22]. Such active targeting moieties include antibody-, aptamer- and folate-based targeting, as summarized in Figure 1.

Due to their favourable specificity and small particle size, peptides are the natural choice for targeting ligands [24,25]. Targeting ability of the 4EBP peptide, which targets the eukaryotic translation initiation factor 4E (eIF4E), has been successfully exploited in OC in vivo [26], as has the OA02 peptide, which has been employed to deliver polymeric micellar NPs to α-3 integrin receptors [27]. Despite this specificity, limitations such as enzymatic degradation leading to their metabolic instability hinder their use [28]. Efforts to circumvent these limitations through modification of the native proteins with the intention of increasing resistance to degradation have been conducted successfully by Dharap and co-workers [29]. Briefly, researchers modified the native sequence of Luteinizing hormone releasing hormone (LHRH) peptide to provide a reactive amino group specifically on the side chain of a lysine residue to yield the super active, degradation-resistant Lys-6-des-Gly-10-Pro-9-ethylamide LHRH analog [29]. Apte and co-workers [30] successfully implemented a strategy to circumvent the degradation of the transactivator of transcription peptide (TATp). TATp is a cell penetrating peptide that aids in the cellular uptake and transportation of micellular and liposomal nanocarriers into cells. This protein is susceptible to proteolytic enzymatic degradation from the acidic tumour environment which reduces its targeting performance in vivo. Success was achieved by shielding the peptide using a polyethylene glycol–Hydrazine–phosphatatidylethanolamine (PEG-Hz-PE) conjugate, bound via a pH sensitive bond that remains stable under normal pHs, shielding the TATp, whilst degrading at acidic tumour pHs facilitating polymer hydrolysis and exposure of the TATp for cellular internalization [30].

### 2.1. Antibody Based Targeting in OC 

Antibodies are one of the major moieties employed as targeting agents for nanocarrier based precision drug delivery systems [23]. Since their introduction, great developments have been made in this field. HER2 (also known as ERBB2 or NEU) is a transmembrane glycoprotein receptor that exhibits tyrosine kinase activity and extensive homology to the epidermal growth factor [31,32]. It is ubiquitously expressed in tumours and known to be overexpressed in approximately 30–40% of all ovarian and breast cancers [32], with considerable variation [31]. HER2 thus presents itself as a promising receptor to exploit for antibody mediated therapy [5].

Trastuzumab, a monoclonal antibody (mAb) was the first HER2 targeting agent evaluated resulting in a poor 7.3% response rate in phase II clinical trials utilizing HER2 positive cancer patients. In 2006, the anti-HER2 antibody Pertuzumab underwent clinical evaluation in OC, therapeutically benefiting 14.5% of patients [33]. Since then, studies have been undertaken to maximize the effects of these mAbs. Palanca-Wessels and co-workers [34] obtained favourable results for Trastuzumab employing Small interfering RNA (siRNAs) for gene suppression. A biotinylated polymeric nanocarrier conjugated with Trastuzumab served as a platform to deliver siRNA. Results indicate that siRNA successfully accumulated in tumours in excessive amounts with a 70% targeted gene suppression in mice bearing intraperitoneal (i.p.) human ovarian tumour xenografts. This system therefore displays improvements in the anticancer activity of Trastuzumab in contrast to sole treatment [34]. 

SYD985 is a novel HER2-targeting antibody drug conjugate (ADC), attached via a cleavable linker to duocarmycin. This novel antibody displays strong activity towards OC as well as moderate to low HER2 expressions. Menderes and co-workers [5] compared the anti-tumour activity of SYD985 against Trastuzumab emtansine (T-DM1), an ADC (which possess superior clinical activity against naked Trastuzumab due to the HER2 targeting effects of Trastuzumab and the antimicrotubular effect the DM1 portion) in ten primary OC cell lines with HER2 expression.

Results indicate that, in the presence of peripheral blood lymphocytes (PBL), both SYD985 and T-DM1 induce similar antibody-dependent cellular cytotoxicity (ADCC). In contrast, SYD985 were 3–42-fold more cytotoxic in the absence of PBL when compared to T-DM1. Unlike T-DM1, SYD985 were successful in effectively destroying HER2 tumour cells. Furthermore, in vivo studies proved that SYD985 is significantly more active than T-DM1 against HER2 positive epithelial ovarian cancer (EOC) xenografts with moderate to low heterogeneous HER2 expressions [5]. This study provides useful evidence suggesting SYD985 possesses superior potency over T-DM1.

The B7-H3 epitope is expressed on OC cells and cancer initiating cells (CICs) [35]. B7-H3 appears to be expressed in 93% of ovarian tumours after immunohistochemical experimentation demonstrated by Zang and co-workers [36]. B7-H3 is a group of transmembrane proteins with 20–30% common amino acid sequences with other B7-family molecules. It retains 88% of the amino acid sequence with mouse B7-H3 [37]. Studies conducted by Fauci and co-workers [37] revealed that, by decreasing the B7-H3 expression in tumours, it inhibits cell migration and invasion, thus making it a promising molecular target to investigate [38]. 

**Figure 2:**
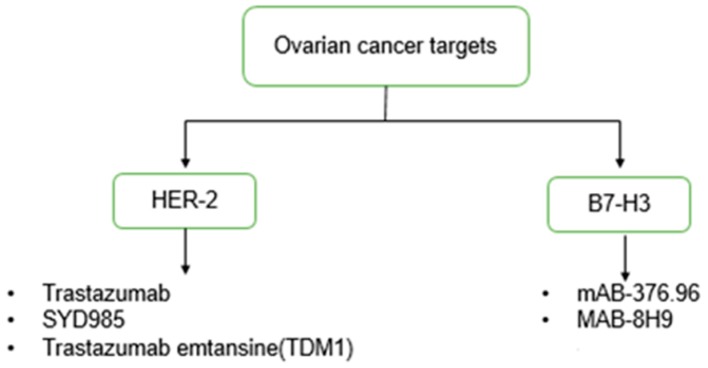
Flow chart representing the two ovarian cancer targets with their respective antibodies [39].

Recently, Kasten and co-workers [35] exploited the B7-H3 epitope for OC intervention using the monoclonal antibody (mAb) 376.96, which recognizes the B7-H3 epitope for α-particle radioimmunotherapy (RIT) in preclinical models of human OC. Survival and bio distribution of the 212Pb-376.96 archetype were compared against an isotype of 212Pb-F3-C25, utilized as a non-targeting control. 212Pb-376.96 inhibited the clonogenic survival of OC cells 40 times more effectively than the control 212Pb-F3-C25. Furthermore, the 212Pb-376.9 system demonstrated high peritoneal retention and tumour tissue accumulation in contrast to healthy tissue [35]. Figure 2 provides a brief summary of the main antibodies that effectively target HER-2 and B7-H3.

### 2.2. Aptamer Based Targeting and Detection in OC

Nucleic acid aptamers have recently gained much attention as an emerging class of active targeting moieties and serve as an impressive bioanalytical tool for the medical field [40]. Aptamers are short single-stranded DNA or RNA oligonucleotides that are folded into secondary and tertiary three-dimensional (3D) structures allowing binding to particular molecular targets with high affinity, specificity and precision. Aptamers commonly bind to proteins but may also bind to nucleic acids, small compounds or entire cells. Although often protein based [23,40,41,42,43], aptamers also comprise of metal ions, drugs and entire cells or viruses [44]. In contrast to the animal derived antibodies, aptamer synthesis employs a Systematic Evolution of Ligands by Exponential enrichment (SELEX) technology [40]. Because of this chemical synthesis, aptamers offer improved stability when compared to the conventional antibodies and enzymes [45]. Furthermore, the flexible nature of aptamers facilitates conformational changes within their structure upon binding to their targets, thus making their use in biorecognition of proteins, cancerous cells, toxins, drugs and other molecules highly favourable [45].

Aptamers possess several advantageous traits over conventional antibodies [44], which were previously regarded as the gold standard approach for detection of proteins [46]. Aptamers achieve high batch to batch uniformity for any given target, whilst excluding the need of biological models for their production [44,47]. Aptamers can also be selected against toxic or non-immunogenic targets with the further benefit of ease of characterization over conventional antibodies. In addition, aptamers are not immunogenic and, due to their small size, generate greater tissue penetration. Different from antibodies, they are sufficiently stable, resistant to harsh environments (pH and temperatures) and can be simply modified to further enhance their stability, bioavailability, and pharmacokinetics [48]. Thus, aptamers are a promising targeting moiety for precision drug delivery. This section of the article will review the latest advances in aptamer targeting approaches for diagnostic and treatment in OC. 

#### 2.2.1. Aptamers in OC Diagnostics 

Early identification of neoplastic diseases is vital for the prompt initiation of therapy, particularly for rapidly metastasizing OC [40]. CA125 is a repeated peptide epitope of mucin, and is implicated in the proliferation of cancer cells, currently becoming the best and most commonly employed serum based biomarker for OC [49]. Previous methods employed to detect CA125 include a variety of immunological and biochemical methods such as radioimmunoassay, enzyme-linked immunosorbent assay (ELISA) electrochemiluminescent immunoassay, chemiluminescence, electrochemical, piezoelectric immunosensors and field effect transistors. These methods are flawed by high cost, long analysis time, sophisticated instrumentation and low levels of sensitivity to detect tumours at an early stage, thus making aptamers a promising candidate for CA 125 detection [49].

Lambertia and co-workers [40] recently developed two nuclease resistant RNA aptamers (CA125.1 and CA125.11) using protein-SELEX strategy. Binding characteristics were studied using real time (RT) PCR and Surface Plasmon Resonance (SPR) towards CA125, with the intention of developing a new aptamer-based SPR biosensor for CA125 detection. Results by reverse transcription polymerase chain reaction (RT-PCR) suggest that CA125.1 displays stronger binding affinities compared to CA125.11. This was further confirmed using SPR binding experiments. SPR results further highlight that the aptamer/CA125 complex can be easily overturned with different binding conditions such as flow action during the SPR measurements. Consequently, additional efforts on aptamer based SPR bioassays is required to achieve a more stable and reproducible immobilization of the aptamer on the SPR chip [40]. Despite the current limitations, this aptamer provides a useful discovery for future study and development. 

More recently, Hamd-Ghadareh and co-workers [49] developed a complex ultrasensitive antibody-ssDNA aptamer sandwich-type fluorescence immunosensor for CA125 detection in OC. The system is based on a novel signal amplification strategy where carbon dots (CDs) were functionalized with the aptamer. Polyamidoamine (PAMAM)-dendrimers/gold nanoparticles (AuNP) were covalently attached to the CA125 antibody to complete the sandwich assay method. The AuNPs possesses high fluorescence resonance energy transfer (FRET) quenching efficiency and utilized as a FRET reagent, whilst the PAMAM-dendrimers served as a platform to immobilize the CA125 antibody, allowing increased sensitivity and accuracy of the immunosensors [49].

Results indicate the immunosensor can detect an extremely low calculated limit of CA125 when compared to other biosensors, exhibiting very high selectivity toward CA125. Furthermore, the immunosensor successfully achieved selective imaging of these cells [49]. 

**Figure 3:**
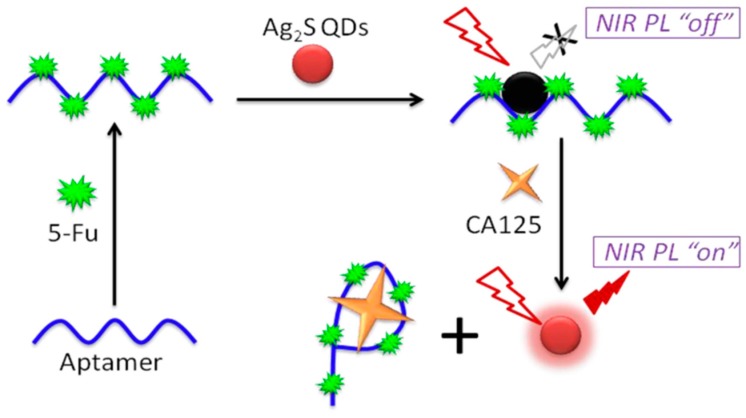
Schematic illustration of the fabrication processes of Ag2S QDs/aptamer/5-Fu hybrid-based near-infrared (NIR) photoluminescence (PL) turn-on probe of CA125. (Reprinted from [45] with permission from Biosensors and Bioelectronics)

Progressing with the use of aptamers in diagnosis for OC, Jin and co-workers [45] developed Ag_2_S quantum dots (QDs) for near-infrared emitting photoluminescence (NIR PL) combined with the aptamer/5-fluorouracil (5-Fu) to produce Ag_2_S QDs/aptamer/5-Fu (Figure 3). 

Results display sensitive NIR PL responses of the probe to the CA125 antigen. Furthermore, the system exhibits high performance in human body fluids including human serum, urine and gastric juice, in addition to high detection recoveries [45]. 

An alternative avenue for OC targeting is the heat shock protein 10 (HSP10). This protein is overexpressed in tissues surrounding the ovaries. HSP10 is implicated in tumour formation due to its role in suppressing T cell activation, allowing the tumour to escape immune surveillance. In addition, HSP10 suppresses apoptosis of malignant cells, thus gaining much recent interest as potential biomarker for early OC detection and clinical diagnosis [50,51]. Chen and co-workers [51] aimed to detect this protein by developing a new protocol for the silane-based surface modification of quartz substrates for the immobilization of hexa-histidine-tagged HSP10, to determine the interaction between the HSP10 protein and a novel DNA aptamer, MND-38, selected through SELEX using electromagnetic piezoelectric acoustic sensor (EMPAS). In this paper, researchers conducted proof of concept work, to show that this method can successfully detect HSP10. This study made use of an electromagnetic piezoelectric acoustic sensor (EMPAS), which was able to achieve extremely high sensitivity. The EMPAS is designed to detect the resonance frequency generated from an electrode-less quartz disc [51]. Researchers in this study were aware of the specificity of the aptamer used, i.e., the MND-38 aptamer via the selection process through SELEX, which was confirmed through DNA–Native PAGE analyses when HSP10 was introduced to the aptamer solution. With properly modified quartz surfaces, aptamer was injected over the surface using the EMPAS. The measurements highlighted that there was a specific interaction between the protein and aptamer when the protein was on the surface. The investigators concluded that the EMPAS is not a gravimetric device, but encompasses a myriad of surface interactions and phenomena that may be questionable for solely quantitative measurements of the protein, and thus may be valuable for qualitative tests to initially confirm when complex interactions are occurring. Furthermore, researchers highlighted the need to investigate the nonspecific binding of the aptamer and the necessity to further optimize the system before testing in clinical samples. It was shown the applicability of this surface in acoustic wave sensors for detecting aptamer binding and allows for future applications towards other His-tagged proteins and corresponding binding partners [51]. This study highlights the significant role aptamers play in HSP10 detection, as antibodies are difficult to produce against this protein, it also highlights the complexity of early OC diagnosis interventions, with potential for greater developments in the field. 

#### 2.2.2. Aptamers in OC Treatment 

Pi and co-workers [52] recently developed a novel a nucleic acid based NP to deliver doxorubicin (DOX) to the overexpressed Annexin A2 protein by incorporating an Annexin A2 A phosphorothioate-modified DNA aptamer, Endo28. The system utilizes a highly robust and thermostable phi 29 pRNA three-way junction (3WJ) as a platform to develop the new therapeutic NPs [52,53,54,55]. Result demonstrate that the Endo28-3WJ possesses stronger binding affinity to IGROV-1 (71.2%) than to SKOV3 (51.7%) (Annexin A2 positive control), with very low affinity for HEK29T (17.3%) (Annexin A2 negative control). These results correlate to the level of Annexin A2 expressions found within the cells [56,57]. In vivo bio distribution studies show accumulation of the NPs in tumours, in contrast to healthy tissue. This comprehensive study highlights the promising targeting capabilities of this aptamer for future OC research and treatment. 

The receptor tyrosine kinase AXL proves to be a promising molecular target to investigate due to its role in cancer cell survival, invasion and metastasis. AXL is overexpressed in ovarian tumours in contrast to healthy ovarian epithelium. Kanlikilicer and co-workers [53] investigated the potential for the AXL aptamer to therapeutically inhibit tumour formation in vitro and in vivo. The AXL aptamer proved to be highly stable, exhibiting good resistance to nucleases and high affinity for AXL with no aptamer localization in non-AXL expressing cells. Therapeutically, the aptamer inhibits AXL activity and reduces OC cell proliferation, further impairing migration and invasion of OC cells, resulting in inhibition of tumour growth and I.p metastatic nodules.

Despite the above reviewed studies, the recent use of aptamers for treatment purposes has been limited since 2016 [52,53], as compared to the use of aptamers in pancreatic cancer (PC). Recently, much work on aptamers in PC treatment has shown promising advances [58,59,60]. Is it therefore possible to apply the techniques employed for the aptamer mediated treatment in PC to OC? This is an important question to consider for the future developments of aptamers in OC treatment [61].

### 2.3. Folate Receptor Targeting in OC

Folic acid (FA) has become increasingly significant as a targeting moiety for the localized delivery of chemotherapeutics to specific cancer tissues and sites of inflammation [62]. FA is an atypical cell-targeting agent which possesses high affinity for its corresponding overexpressed folate receptor (FR) [63]. A study conducted by Garin-Chesa and co-workers [64] to determine the frequency of FR overexpression in various tumours revealed that 52 out of 56 OC cases presented with FR overexpression [21]. Extensive studies on the FR [65] enabled researchers to conjugate antineoplastic drugs, antisense oligonucleotides, protein, toxins, and imaging agents with FA for targeting purposes [66].

Recently, Lv and co-workers [65] developed a Capsaicin (CAP)-loaded FA-conjugated lipid NP for OC intervention. Results confirmed a higher cellular uptake were obtained in the FA targeting system when compared to the non-targeting system, in addition to higher toxic effects and cellular apoptosis.

FA conjugation was further employed for the precision delivery of DOX and docetaxel (DTX)-conjugated poly (lactic acid)-poly (ethylene glycol)-folate (PLA-PEG-FOL) based polymeric micelles for controlled release OC combination therapy, as a strategy to reduce resistance. In vitro cellular uptake studies of the polymeric micelles revealed that DOX, FA-conjugated DOX and non-targeting PLA-PEG micelles were 52%, 32.8% and 16.8%, respectively [67].

Cytotoxicity results revealed that both DTX and DOX targeted polymeric micelles exhibit greater cytotoxic effects in contrast to the non-targeting micelles. These two polymeric micelles display promising outcomes in targeting the overexpressed FR and desirable drug release in acidic environments, to produce an improved in vitro cytotoxic performance to circumvent resistance [67]. Alberti and co-workers [68] demonstrated a novel theranostic polylactic and polyglycolic acid (PLGA) NP to deliver boron (B)-curcumin complex (RbCur) and gadolinium (Gd) complex into tumour cells for B and Gd neutron capture therapy (NCT), whilst Gd allowed for magnetic resonance imaging (MRI). 

Results confirmed that the FA-targeted NPs displayed stronger affinities for the FR, with negligible cell binding for the non-targeted NPs [68]. Furthermore, cells treated with PLGA-NP-FA irradiated with neutrons (Gd-BNCT-group) show lower percentage of viable cells in contrast to the cells treated with the currently clinical investigated BNCT drug l-para-boronophenylalanine (BPA) (BNCT group). This indicates that the presence of Gd in conjunction with FA conjugation produces successful anticancer outcomes when compared to sole BNCT. In addition, curcumin benefited the system by decreasing cell proliferation. The system effectively measured Gd and B concentrations indirectly by MRI, opening new perspectives for neutron capture applications [68]. 

### 2.4. Is the Active Targeting Avenue Alone a Way Forward for Cancer Nanomedicines?

The active targeting avenue presents recent success in cancer therapies but faces several crucial limitations. Challenges, such as heterogeneous blood flow, Large intervascular distances, long interstitial path lengths, slow transportation rates and differences between tumour structure and composition of various cancers, hinder their direct tumour targeting abilities. In addition, upon exit from blood vessels, these targeting systems usually bind to cells they first interact with on the periphery, possibly healthy cells, hence the need for targeting systems to not solely target overexpressed receptors but rather receptors that are in cancerous tissue specifically [69]. The ultimate cancer nanomedicine should be formulated such that targeting, distribution and anti-cancer performance is independent of the inherent bodily processes. To achieve this, a complete understanding of tumour microenvironment and vasculature in addition to the route the nanosystem must travel is necessary. To this end, sole active targeting approaches may require additional aid of passive targeting interventions as a combination strategy for cancer drug delivery.

## 3. Nanocarrier Based Delivery in OC 

Nanocarriers are extensively employed in the pharmaceutical industry for their biomedical applications including: imaging, diagnosis, and delivery of chemotherapeutic [70,71,72,73,74,75,76]. Their versatility ensures an optimal drug release, maximizing therapeutic efficacy. Various carriers used for drug delivery include polymers, quantum dots, nanospheres, nanogels, liposomes, micelles and the more recent magnetic NPs [77,78]. Nanocarriers create an avenue to overcome various limitations, e.g., increasing the bio distribution of potent drugs at the target site whilst minimizing system exposure [79]. Furthermore, these carriers provide rate-controlled drug delivery, allowing predictable concentrations and clearances [80]. In cancer, NPs are exploited, as they ensure increased penetration of blood capillaries in cancerous tissue by enhanced permeation and retention (EPR) resulting in increased drug concentrations, in addition to providing an ideal platform for active and passive targeting interventions [81,82,83,84].

Many reviews have previously highlighted the nanocarriers employed in OC [16,85]. In this updated review, we will focus specifically on the most recent and novel nanocarrier based systems targeting OC. 

### 3.1. Nanocarriers for the Delivery of siRNAs to OC

**Figure 4:**
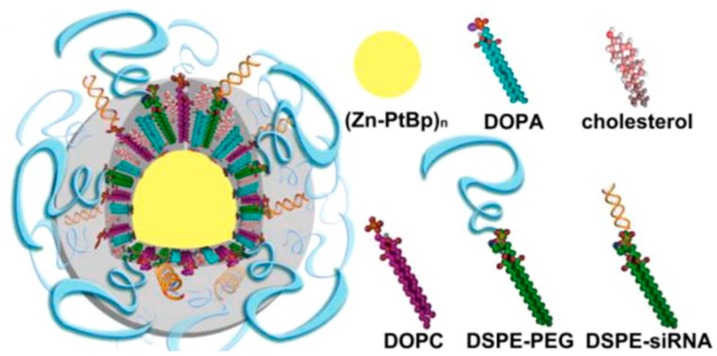
Schematic representation of NCP-1/siRNAs carrying cisplatin in the solid core and siRNAs in the lipid shell. Reprinted (adapted) with permission from [86]. Copyright (2016) American Chemical Society.

siRNAs play a key role in drug resistant OC due to their ability to disrupt cellular multiple drug resistant (MDR) pathways by silencing the expression of relevant genes including *MDR* genes and overexpressed receptors [86]. Their discovery has revolutionized the field of gene-based therapies, providing greater opportunities for successful cancer treatments [87]. The effective delivery of siRNAs relies heavily on the appropriate nanocarrier system as native siRNAs following systemic administration fails to exhibit features required for movement through cellular membranes. Native siRNAs are also susceptible to degradation by serum nucleases and rapid renal clearance [88]. Despite previous success of nanosystems to increase the therapeutic potential of siRNAs, the optimal delivery of siRNAs requires a more “tailor made” approach to overcome these limitations [3,86,89]. He and co-workers [86] produced a novel core-shell nanoscale coordination polymer (NCP) NP for the combination delivery of cisplatin and cisplatin plus gemcitabine (in the core), whilst siRNA resided in the lipid layer (Figure 4). This system is intended to down regulate the *MDR* gene expression in drug resistant OC.

The smart release of the siRNAs exploited the addition of cisplatin, in contrast to the conventional cationic excipients that are generally required for the effective endosomal escape of siRNA. It was hypothesized in the study that the carbon dioxide (CO_2_) obtained from cisplatin will disrupt the membranes facilitating siRNA release. This was confirmed by research using confocal laser scanning microscopy (CLSM). The system further displayed enhanced blood circulation of siRNA to provide long lasting tumour eradication in addition to increased tumour uptake. Overall, effective therapy was obtained in mouse models with cisplatin resistance OC [86]. Thus, this system offers a promising direction for the delivery of siRNAs to cancer. 

HuR is a ubiquitous protein that is highly expressed in OC and correlates to poor prognosis [89]. Huang and co-workers [89] employed a novel derivatized DNA dendrimer (3DNA) as a nanocarrier for the effective delivery of siHuR (combination of siRNAs against HuR) to target the HuR in nude mice. Targeting was achieved via the use of FA conjugation to produce the FA-3DNA-siHuR system. The conjugation of siHuR to 3DNA retained its ability to suppress HuR expression, following the in vitro transfection of A2780 cells. Therapeutic efficacy was studied in OC mouse models indicating suppression of tumour growth, although not significant, and an increase in survival times for mice. In addition, the FA targeting system improved both efficacy and life span outcomes in contrast to the non-targeting system. It was concluded from the study that the system employed systemically administered siHuR to inhibit HuR expression to achieve desirable therapeutic outcomes [89]. Table 1 provides a summary of the siRNAs employed in OC intervention. 

### 3.2. Nanocarrier for the Delivery of OC Drugs

Hydrophobic chemotherapeutics have been incorporated into advanced self-assembling amphiphilic NPs. The hydrophobic region acts as a reservoir to house the poorly soluble drug, whilst the hydrophilic region offers protection against the removal by the reticuloendothelial system. Employing a pH stimuli responsive linker to the drug enables release at specific conditions and predetermined rates, a factor necessary for control release purposes [95,96]. Theranostic approaches provide the perfect combination for treatment and detection and recent work conducted by Lin and co-workers [24] intended to produce such system [97].

Hyaluronic acid (HA) NPs were employed as self-assembling nanocarriers for the targeted delivery of DOX coupled with a NIR dye for theranostic application in OC. LHRH served as targeting peptide to actively target the overexpressed corresponding LHRH receptor [98,99]. Controlled drug release was achieved via a stimuli responsive spacer, using cis aconityl linkage and a disulphide bond that possess pH sensitive and redox properties respectively. The cis aconityl linkage allowed cleavage of DOX in acidic environments (pH 7.5–5.5) and the disulphide linkage enhanced its stability at low extracellular glutathione concentrations whilst at high glutathione concentrations facilitated drug release [24]. Cytotoxicity studies revealed that high uptake and therapeutic activity was obtained in cell lines overexpressing the LHRH receptors, with lower toxic effects in non-receptor expressing cell lines. Furthermore, the LHRH-conjugated NPs possess desirable tumour imaging capabilities and an excellent anticancer effect, such that almost 30% of original tumour size was reduced in 20 days [24]. This “Environmentally sensitive” system exhibits excellent performance in orthotropic ovarian tumour models possessing greater efficacy over conventional drug delivery systems due to its stimuli responsive nature [100]. 

Kulhari and co-workers [25] successfully improved the delivery of Gemcitabine hydrochloride (GEM) towards OC. GEM is a water soluble, clinically approved anticancer drug that encompasses limitations including short plasma half-life, degradation in body fluids and severe haematological side effects, rendering the drug unsuitable for intravenous injection. To achieve enhanced delivery, a polymeric drug delivery system synthesized using PLGA NPs was developed. 

Cyclic RGDfK (cRGDfK), a five-amino acid peptide, was selected as an active targeting ligand, due to its favourable characteristics including high stability and specificity for the overexpressed αvβ3 integrin receptors observed in OC [101,102,103]. Following synthesis and characterization, results demonstrate a narrow size distribution, high encapsulation efficiency, sustained drug release, increased biocompatibility and efficient uptake of the nanoconjugates were obtained. Furthermore, the anti-proliferative activity of GEM was enhanced via intracellular processes including reduction mitochondrial membrane potential, increased intracellular reactive oxygen species levels and promotion of apoptosis [25]. 

Following blood compatibility studies, encapsulated GEM was able to overcome the haemolysis effect to a considerable extent over native GEM as direct contact to RBCs was reduced, a highly successful outcome for the future safe clinical use of GEM [25]. Table 2 summarizes the Nanocarriers involved in delivery of cancer therapeutics to OC.

### 3.3. What Happens to These Nanosystems Beyond the Realms of Research?

Despite the recent advances in nanocarriers mentioned in this review, the goal of anticancer nanomedicines research should ultimately intend to produce a product that can be clinically beneficial. How big or small is the gap between the latest and cutting-edge research and final delivery to patients? A comprehensive review recently written by Lisa Bregoli and co-authors [107] highlighted the nanocarriers, e.g., liposomes, micellular and polymeric drug delivery systems, that have made it to clinical approval as well as the nanosystems undergoing clinical trials. Unfortunately, the majority of the research rarely makes it to clinical approval. In addition, the movement of nanosystems to clinical use is much lower compared to native small molecule drugs.

The paper highlights the major scientific aspects that hinders the movement of nanomedicines to commercialization, such aspects can be summarized as follows: (1) the complexity of the cancer microenvironment makes understanding of the in vivo distribution of NPs difficult, which is a problem associated with both active and passive targeting; (2) safety and toxicity concerns, and the need to establish better models to determine safety that will best mimic human like behaviour; (3) the lack of physiochemical characterization of NPs and its surface ligation, which is of paramount importance for correlating biological toxicological consequences and biological responses; (4) large scale manufacturing with high batch to batch uniformity to maintain high product quality is necessary, with concerns related to the lack of appropriate methods to test impurities, contamination and aggregation; and (5) the quantification of the API encapsulated within this nanosystems to ascertain dosage requirements [107]. To this end, the translating of well working nanomedicines from the literature, to meeting all requirements necessary for commercialization encompasses a great deal of complexity.

Cheng and co-workers [108] highlighted that aspects such as safety, environmental and regulatory aspects are still key issues hindering the forward movement of recent literature to market. Further describing how regulatory requirements, environmental sustainability across product lifecycle, scientific information, safety of manufacturing materials, scientific uncertainties and scientific risks collectively hinder the movement of these research interventions to market.

In conclusion, the majority of the systems developed in the literature rarely make it to market as the gap between current therapy developments and hospital treatment remains evident. The development cycle of nanomedicines should ideally cover novel, innovative and cutting edge technological advances whilst not neglecting its potential fate in hospital use, employing pre- formulation approaches that ensure its future clinical use. Nevertheless, it is a highly appreciated that producing novel research is a challenging task with further complexity involved for commercialization.

## 4. Glycosaminoglycans as a Potential Molecular Target to Stop the Progression of OC 

In the past, most OC targeting initiatives focused primarily towards the sensitive and highly specific biomarkers on the tumour cells, including the currently exploited CA125 glycoprotein [109]. Most OC initiatives address components such as the cell surface (glycol), proteins and overexpressed receptors, neglecting the potential of the tumour cell surroundings (extracellular matrix). This especially applies for the information-dense class of glycosaminoglycans (GAGs) [110]. 

The ECM forms a vital component of cells and vascular biology. It is responsible for structure and stability [111]. The ECM is a critical environmental determinant of tumour cell behaviour. The matrix serves as a scaffold for the tumour cells to adhere, migrate and proliferate and allows interactions with cellular receptors, growth factors as well as cytokines [110,112]. 

In healthy individuals, proteoglycans (PGs) are present within ECM. PGs are vastly anionic macromolecules that are particularly important in cells as they are responsible for maintaining cellular integrity and function [113]. Core proteins and unbranched polysaccharides covalently bind to form PGs; these polysaccharides are termed GAGs [114]. Based on the monosaccharides and their sulphation patterns, various groups of GAGs occur, including chondroitin sulphate (CS), dermatan sulphate (DS), heparin Sulphate (HS), keratin sulphate and HA [110,115]. Furthermore, these GAGs are implicated in the process of carcinogenesis, including tumour growth, migration, invasion, metastasis and pathological angiogenesis, making them an attractive class of sugars to target [110].

### 4.1. Chondroitin Sulphate as a Molecular Target 

CS is a negatively charged water soluble polysaccharide comprising of repeated units of d-glucuronic acid and d-*N* acetyl galactosamine. CS can be sulphated at various positions, i.e., C-4 and/or C-6 or C-2 of the *N*-acetylgalactosamine and the glucuronic acid moiety, respectively [113]. CS differentiates into five isoforms based on the specific sulphation pattern, giving rise to molecules such as CS-A (GlcA-GalNAc-4-sulphate), CS-C (GlcA-Gal-NAc-6-*O*-sulphate), CS-D (GlcA(2-*O*-sulphate)–GalNAc(6-*O*-sulphate)), CS-E (GlcA-GalNAc-(4,6)-*O*-disulphate) and CS-B (DS) (IdoA (2-*O*-sulphate)–GalNAc(6-*O*-sulphate)). These sulphation patterns dictate specific interactions with various growth factor including Fibroblast growth factor (FGF), hepatocyte growth factor (HGF), Transforming growth factor (TGF) and vascular endothelial growth factor (VEGF) [116]. In healthy cells chondroitin sulphate proteoglycans (CSPGs) have been identified to control aspects of cellular behavior and function [117]. As early as 2003, it was well recognized that these PGs and their carbohydrate residues mediate many tumour cell functions; however, their exact roles in cancer metastasis was poorly established. Since then, recent discoveries and advances reduce this knowledge gap and widen our understanding of GAGs and its contribution to OC. 

#### 4.1.1. The Role of CS in OC

The implication of CS-E to OC has been highlighted in a paper that was previously published from our group [115] and is summarized in Table 3. This section of the article aims to further motivate the potential of CS-E as a potent molecular target. Many studies have reported the elevation of CSPGs levels in tumours, compared to non-malignant tissue. Some relationships have been proposed between tumour GAGs and tumour-cell properties [118,119].

Elevated CS-E levels have been reported in the ECM of OC, particularly in the stromal region, and correlate to the aggressiveness of tumours [115,120]. This stromal component is responsible for the vicious tumour progression and metastasis of OC [116]. 

Based on the above evidence, CS-E proves to be a major culprit in OC development and displays promising potentials for targeting. By inhibiting CS-E activity employing drug therapy, offers a new perspective for clinical treatment and diagnosis.

#### 4.1.2. Targeting Chondroitin Sulphate-E 

Despite previous targeting of cancer cells, the positive impact of tumour-cell targeting vs. non-targeting mechanisms proves unsatisfactory [19,125]. Most of these studies overlook the cancer ECM as a promising alternative for drug targeting. The largely neglected ECM provides several potential molecular targets for precision therapy of chemotherapeutics [126]. Recent studies demonstrate that the delivery of anti-cancer drugs to the tumour stroma significantly eliminate cells and their micro-environment in vivo [127,128]. Furthermore, cellular target expressions may change over time resulting in resistance, posing additional challenges [126]. Therefore, more focus on targeting the ECM may be an important breakthrough for OC [126]. Recently, CS-E was established to be overexpressed in OC ECM and made novel molecular target for anti-cancer therapy [115,129].

In the past, Yamagata and co-workers [130], Smetsers and co-workers [79] and ten Dam and co-workers [124] established the ability of the MO-225, IO3D9 and GD3G7 antibodies to recognize CS-E respectively (Table 4). Problems associated with these antibodies were recognized. Despite the ability of the GD3G7 antibody to bind to CS-E, the antibody also displays specificity to CS-A and CS-H abundant H-units (doAβ1-3GalNAc (4S, 6S)) after analysis using phage display technology directed against rat embryo [124]. IO3D9 possesses weak binding affinities for CS-A or CS-C. Thus, in the past, no antibodies recognize CS-E specifically, increasing the demand for a CS-E specific antibody. Table 4 summarizes the various antibodies that target CS.

In 2015, Watanabe and co-workers [129] discovered two novel CS-E specific monoclonal antibodies, E-12C and E-18H. These antibodies were obtained by digesting CS-E fractions rich in the E subunits with hyaluronidase, susceptibly reacting with low sulphation moieties of the CS molecule. To rule out cross reactivity between 6-*O*-sulphated CS-A and DS that possessed ∼60% of GalNAc (4S, 6S) moieties in their structures, surface plasmon resonance assay with CS-E and CS-A were conducted, suggesting a strong correlation with the molecular weight of CS-E and the E-unit content of 6S-CS-A and providing evidence that these antibodies recognized CS-E specifically. To further verify this, E-12C and E-18H antibodies were reacted with an artificial CSH from DS possessing (IdoAβ1-3GalNAc (4S) resulting in no reacting ability of either antibody, concluding that these antibodies strictly recognize the E-unit (GlcAβ1-3GalNAc (4S, 6S)). Hence, these antibodies can distinguish the structural difference between GlcA and IdoA in the GAG molecules [129]. An important breakthrough for future of targeted therapy considerations.

**Figure 5:**
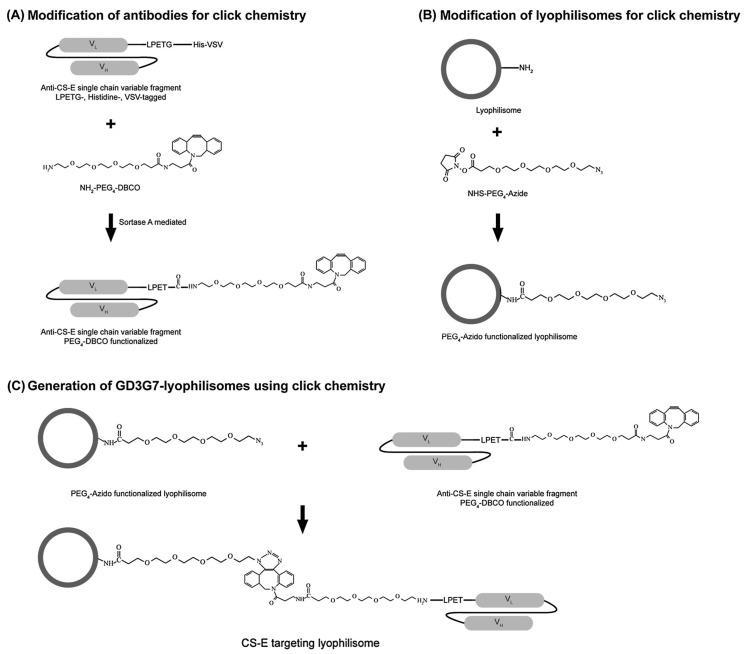
Construction of cancer targeting lyophilisomes depicting conjugation between albumin-based lyophilisomes and GD3G7 antibodies reactive with CS-E, by applying a two-step approach comprising sortase mediated ligation and click chemistry. (**A**) LPETG-His-VSV tagged single chain GD3G7 antibodies were modified for click chemistry by introducing amino-PEG4-DBCO through a reaction mediated by Sortase A. (**B**) Lyophilisomes were functionalized for click chemistry by conjugating PEG4-azide to the primary amine groups of lyophilisomes mediated by *N*-hydroxysuccinimide (NHS). (**C**) Antibody-functionalized lyophilisomes were generated by a click reaction between azido-conjugated lyophilisomes and GD3G7-PEG4-DBCO antibodies. Abbreviations: CS-E, chondroitin sulphate E; DBCO, dibenzylcyclooctyne; VL, light chain variable domain; VH, heavy chain variable domain; SrtA, Sortase A. (Reprinted from [126] with permission from Elsevier)

Van der Steen and co-workers [126] recently formulated GD3G7 functionalized lyophilisomes to precisely deliver DOX to the tumour stroma of OC. This was achieved via a two-step approach involving sortase-mediated ligation and bioorthogonal click chemistry (Figure 5) [126]. In vitro, the lyophilisome released a substantial amount of DOX leading to cell death. The lyophilisomes also showed superior cell viability reducing properties and entrapment in contrast to liposomal DOX. Furthermore, the GD3G7-lyophilisomes displayed strong reactivity in CS-E producing cell, in contrast to the non-targeting control. Specificity studies on the GD3G7-lyophilisomes suggest that high affinity for CS-E is obtained, with background signals for other immobilized GAGs. This study highlights the advantage of targeting the ECM, in addition to the niche design of suitable nanocarriers for enhanced anticancer performance [126].

The GD3A11 antibody selected through phage display technology by van der Steen and co-workers [120] were used to investigate the potential of highly sulphated CS (CS-E) and the CSPG versican, as a biomarker in OC. Results revealed that the GD3A11 epitope was minimally expressed in normal organs. Intense expressions were noted in the ECM of different OC subtypes, relative to benign ovarian tumours. The expressions were independent of tumour grade and FIGO stage. Furthermore, no mutational change in methylation status was observed for the *CHST15* gene. The GD3A11 antibody showed strong reactivity for CS-E subtype rich in GalNAc4S6S disaccharides [120]. The GD3A11 antibody displays favourable specificity to CS-E exclusively, an important trait to consider for the future use of this antibody in diagnostic and treatment purposes.

### 4.2. Hyaluronan (HA) As a Potential Molecular Target 

Similar to other GAGs, HA is an extracellular anionic polysaccharide [131,132]. HA is non-sulphated and consists of repeated units of d-glucuronic and *N*-acetylglucosamine [131]. HA is ubiquitously expressed in the ECM of various tissues including connective, neural, epithelial and rapidly growing foetal tissues, in contrast to mature adult tissues [132,133,134]. The biosynthesis of HA is facilitated by the plasma membrane protein; HA synthase (HAS), which have several isoenzymes i.e., HAS1, HAS2 and HAS3, with active sites present on the intracellular surface of the membrane [131]. HA degradation is induced by a family of six hyaluronidases (Hyal), consisting of Hyal1, Hyal2, Hyal3, Hyal4, PH-20/PSAM1 and the pseudogene *HyalP1* [135]. HA is responsible for regulating cellular activities such as proliferation, migration and infiltration [132]. 

#### 4.2.1. The Role of Hyaluronan, CD44, Hyaluronidase and Hyaluronan Synthase in OC

Cluster of differentiation 44 (CD44) is a group of multifunctional trans-membrane acidic glycoproteins. It is particularly important to OC progression as it serves as a surface receptor for HA binding at the N terminus of the extra cellular domain [3]. HA is the primary ligand for CD44 which is overexpressed in OC, causing cell adhesion, migration, invasion, proliferation, and angiogenesis [3,136]. Furthermore, this binding initiates the direct cross-signalling between different signalling pathways including HER2, Src kinase and ERK. CD44 is proposed to be involved in increasing the motility of cancer cells and differentiation of cancer stem cells [91,137]. Co-overexpression of CD44 and MDR1 and MDR2 proteins correlate to OC progression [138]. In addition, it has been proposed that CD44 positive OC patients show a remarkably lower survival when compared to negative CD44 tumours [3]. 

Metastatic tumours, including breast tumours are frequently enriched with HA [139]. HA mediates the action of physical linkage between CD44s and Her2/ErbB2 tyrosine kinase, which results in the rapid stimulation of ovarian carcinoma cell growth [140]. When CD44-siRNAs are delivered to ovarian tumours it down regulates CD44 resulting in prevention of HRG-mediated ovarian tumour cell growth and decreased migration [141]. 

Hiltunen and co-workers [142] analysed the HA concentration and Hyal activity in OC. Quantitative HA assays indicate a statistically significant increase in HA levels occurs in malignancies in contrast to normal tissues, functional cysts, borderline and benign tumours. This elevated HA concentration provides an additional growth advantage for primary ovarian tumours. Hyal levels were found higher in borderline and benign epithelial ovarian tumours, as compared with functional cysts but remained low in the malignant epithelial tumours [142]. 

Despite the large pool of information, unclear results question the defining roles of HA-synthesizing and HA degrading enzymes in OC. With respect to Hyal, it was found that high activity was reported in malignant ovarian tumours compared to endometrial and cervical tumours [143], but reported to be lower in another study when compared to benign and borderline tumours of the ovary [142]. Weiss and co-workers [144] performed a study that intended on establishing the expression and clinical role of the various HAS and Hyal enzymes and their splice variants in serous OC effusions.

HAS1 mRNA were overexpressed in effusions, in contrast to primary carcinomas and solid metastases, whilst HAS2 mRNA was overexpressed in solid metastases and primary carcinomas compared to effusions and HAS3 mRNA was overexpressed in all subtypes. Similarly, the Hyal family also displayed varying degree of expression with respect to tumour subtype, however HYAL1 was uniformly absent. The latter finding agrees with the data obtained by Nykopp and co-workers [145]. Furthermore, HAS1 mRNA was overexpressed in pre- compared to post-chemotherapy effusions, with opposite finding for HYAL2-var1 and HYAL3-WT. These data suggest that both enzymes, excluding Hyal1, are present in fluctuating concentrations with respect to tumour type, with HAS1 being associated with the more aggressive subtypes. These results highlight the complexity of these enzymes and their roles in OC [144]. The implications of HA and CD44 to OC is further summarized in Table 5.

#### 4.2.2. Targeting Hyaluronan 

HA conjugated to chemotherapeutics, e.g., paclitaxel (PTX), has been introduced previously to enhance the targeting of therapies [138,144]. Hyaluronic acid (HA) provides favourable traits for the prodrug approach where the drug-HA conjugate becomes active upon release. Several of these conjugates are observed due to HA possessing several functional groups facilitating drug conjugation. In addition, HA has been ligand onto drug loaded NPs for enhanced targeted delivery [146]. Furthermore, HA offers physiological and biological advantages such as biocompatibility, biodegradability and non-immunogenicity characteristics. Currently, HA has been employed in various drug delivery systems including nanocomplexes in addition to NPs and drug conjugates as targeting agents [99].

Previous in vivo anti-tumour activity of HA-based drug delivery systems have been reported. However, recent advances show promising results for HA in OC targeting [147]. 

Yang and co-workers [3] designed a self-assembling HA-PEI/HA-PEG NP targeting CD44 receptors to deliver MDR1 siRNAs to circumvent MDR OC. Results support the ability of the system to actively target and effectively delivery MDR1 siRNA to MDR OC in vitro and in vivo. Furthermore, HA-PEI/HA-PEG NPs down regulated the expression of MDR1 and increase MDR cellular sensitivity of PTX. Cellular uptake and encapsulation efficacy of siRNA were also favourable [3]. This study provides a highly promising perspective for future HA targeting in OC treatment. Montagner and co-workers [136] developed a bioconjugate composed of HA and SN-38, an active metabolite of the FDA approved anticancer drug irinotecan, to target the CD44 receptor in OC. Positive interaction of the bioconjugate and CD44 receptors were obtained in addition to improved therapeutic efficacy compared to native irinotecan. The SN-38 conjugation to HA enhanced the in vivo tolerability and broadened the therapeutic application of irinotecan. To conclude, this approach offers a promising approach for the loco-regional treatment of OC.

## 5. Conclusions and Outlook 

This article intends to highlight the recent advances in OC treatment and diagnosis, introducing the novel and innovative technologies currently developed. The majority of studies exploited various active targeting mechanisms to provide potent, site specific delivery of chemotherapeutics to specific overexpressed molecular targets. Coupling this with a suitable vehicle provides a platform to notably: (1) reduce the toxicity of native drugs towards healthy cells; (2) enhance circulation of native drugs; (3) increase efficacy of treatment; (4) improve cellular uptake of native drugs; and (5) increase spatial localization of drugs at target sites, thus rendering effective clinical responses less reliant on the pharmacological activity of conventional chemotherapeutics. This means of formulation development creates avenues to detour previous limitations of native drug treatment, e.g., insolubility, whilst providing potent therapeutic effects. Studies reviewed in this paper highlighted a collective movement in OC initiatives, with further room for optimization before their application in clinical treatment. The discovery of novel biomarkers and targeting ligands becoming increasingly studied aid the formulation development process, particularly for targeted delivery, that has displayed some success thus far. 

The second half of this paper reviewed GAGs as potential molecular target, with the intention of providing comprehensive evidence that encourages more research and treatment strategies aimed towards CS-E and GAGs. Employing NPs loaded with drug molecules that specifically target GAGs creates the potential for the development of a novel class of cancer therapy termed “GAG inhibitors”. Although these advances provide useful treatment/diagnostic platforms, OC is still a highly complex disease with the need for further research interventions to ensure safe, efficacious and cost effective future medicines.

## Figures and Tables

**Table 1:** Presenting the various nanocarriers involved in siRNA delivery to target OC and the systems response.

Drug	Targeting Moiety	Nanocarrier	Response	Reference
Cisplatin, GEM and siRNA	-	Lipid based nanocarrier, with a self -assembled core-shell NCP	- NCP-siRNAs NPs efficiently downregulated the *Bcl-2* gene expression in SKOV-3 and A2780/CDDP cells by >70%. - NCP-siRNAs NPs successfully eradicated tumours causing 100% survival in mouse models for > 90 days. - 92 days after tumour inoculation, NCP-1/siRNAs treated mice were sacrificed with no evidence tumours.	[86]
siRNA	FA to target HuR Overexpression	Derivatized DNA dendrimer	- When Mice were injected twice weekly with FA-3DNA-siHuR for 4 weeks, the median survival of FA-3DNA-siHuR-treated mice were approximately 1.5 times longer than the controls.	[89]
Co-delivery of cisplatin and siRNAs	-	NMOFs	- The cisplatin IC_50_ values of free cisplatin, UiO-Cis, pooled siRNAs/UiO-Cis, free cisplatin plus free pooled siRNAs, and free cisplatin plus pooled siRNAs/UiO were 53.9 ± 4.7, 53.2 ± 4.4, 4.7 ± 1.8, 45.1 ± 7.0, and 6.6 ± 0.3 μM, respectively. - Co-delivering of pooled siRNAs and cisplatin employing NMOFs decreased the IC_50_ value >11-fold, in contrast to free cisplatin and UiO-Cis.	[90]
PTX and siRNA	LHRH peptide	Nanoscale PPI dendrimer	- LHRH-PPI-siRNA and LHRH-PPI-PTX combination enhanced the cytotoxicity of the conjugate. - The viability of ascitic cells were decreased almost 10-fold in comparison to the control cells, more than 5-fold when compared with free PTX and more than 2-fold when compared with non-targeted PPI-PTX-siRNA complex. - The combination caused almost complete tumour shrinkage within 28-days.	[91]
siRNA	HA-NP system targeting CD44 receptors	HA based self-assembling NPs	- Tumour volume of mice treated with HA-PEG/MDR1 siRNAs targeted NPs was approximately 3-fold lower than in mice treated with native PTX.	[3]
DOX and siRNA	NA	MSNP	- MSNP with siRNAs caused increased cellular accumulation of DOX. - The IC_50_ value of the siRNA delivering MSNP was approximately 2.5 times lower than the IC_50_ of free Dox or other Dox loaded particles.	[92]
Blc2-siRNA and DOX	FA-targeting overexpressed FR	copolymer self-assembled cationic micelles	- The highest apoptosis of 77.5% were observed in cells incubated with FA-DOX-Bcl2 siRNA-NPs leading to potent synergistic effects inducing cell apoptosis.	[93]
siID4	tandem tumour-penetrating and membrane-translocating peptide to target ID4	TPN	- The tumour burden in mice that received TPN/siID4 remained low compared to controls, 80% of these recipients survived >60 days, despite treatments stopping at day 50. - No visible tumour lesions in 4 out of 5 TPN/siID4–treated mice occurred indicating tumour regression. - Histological analysis revealed significant reduction in ID4 levels and increased apoptosis in the tumour parenchyma of mice treated with TPN/siID4.	[94]

Abbreviations in the table are defined as follows: NMOFs, nanoscale metal−organic frameworks; GEM, gemcitabine; LHRH, luteinizing hormone-releasing hormone peptide; PPI, polypropylenimine; PTX, paclitaxel; CD44, Cluster of differentiation 44; HA-NP, Hyaluronan Nanoparticle; DOX, Doxorubicin; MSNP, mesoporous silica nanoparticles; siID4, ID4-specific siRNA; TPN, tumour penetrating nanocomplex.

**Table 2:** Presenting various nanocarriers involved in drug delivery for OC and the systems response.

Drug	Targeting Moiety	Nanocarrier	Response	Reference
DOX	FA to target FR	pH-sensitive micelles	- The micelle formulation effectively decreased the growth of existing MDR tumours in mice for at least 50 days by three i.v. injections at a 3-day interval at a dose of 10 mg DOX/kg. - Tumour growth rate of the micelle group was delayed when compared with free DOX group.	[104]
PTX	OA02 peptide	Micellar NPs formed using PEG-block-dendritic CA copolymers (PTX-OA02-NPs)	- PTX-OA02-NPs displayed superior tumour growth inhibition than Taxol^®^, at equivalent PTX dose of 10 mg/kg. - The median survival time of mice in the group of PBS control, Taxol^®^, PTX-NPs (10, 30 mg/kg), and PTX-OA02-NPs (10, 30 mg/kg) were 20, 27, 29, 69, 32, and 95 days, respectively.	[27]
PTX and CDDP	NA	Biodegradable, biocompatible polypeptide-based polymeric micelles	- MTT assays on A2780 cells revealed IC_50_ values Free CDDP is 1.5 in contrast to (CDDP + PTX)/cl-micelles of 0.14 exhibiting superior killing properties.	[105]
DOX	TATp	PEG-Hz-PE conjugated immunoliposomes	*-* In vitro cytotoxicity revealed the lowest cell viability was obtained Lipodox-TATp in both SKOV-3 sensitive and SKOV-3 resistant cells yielding IC_50_ values of 0.36 and 3.12 respectively in contrast to lipodox yielding IC_50_ values of 6.25 and 100.00 respectively.	[30]
Co-delivery of PTX and curcumin	TF to target resistant OC spheroids and in vivo tumours	PEG-PE based polymeric micelles	- The TF-targeted PTX system displayed and enhanced micelle penetration into spheroids reducing cell viability to 35.3 ± 2.7% at 6.9 µM of micellar PTX concentrations when compared to free PTX at 80 ± 22% at 6.9 µM dosage.	[106]
GEM	cRGDfK peptide targeting αvβ3 integrin receptors	PLGA based NPs	- The IC_50_ values after 48 hours of incubation in SKOV3 cells for GEM, GEM-NPs and GEM-RGDfK-NPs were 0.572 ± 0.013 μg/ml, 0.148 ± 0.01 μg/mL and 0.034 ± 0.004 μg/mL respectively. - The improved anticancer efficacy may be attributed to the targeting properties of the peptide.	[25]
DOX coupled with a NIR dye for theranostic application	LLHRH	self-assembling HA NPs,	- Results demonstrate that LHRH-conjugated NPs possess a desirable tumour imaging capability and an excellent anticancer effect, such that almost 30% of the original tumour size was reduced in 20 days.	[24]

Abbreviations in the table are defined as follows: PTX, paclitaxel; i.v., intravenous; PEG, polyethylene glycol; CA, cholic acid; CDDP, cis-dichlorodiamminoplatinum; TATp, transactivator of transcription peptide; PEG-Hz-PE, Polyethylene glycol–Hydrazine–phosphatidylethanolamine; TF, Transferrin; PEG-PE**,** polyethylene glycol-phosphatidylethanolamine; GEM, Gemcitabine hydrochloride, PLGA, poly (d,l-lactic-co-glycolic) acid.

**Table 3:** The correlation of CS-E to OC, summarising and supporting CS-E as an important biomarker for OC diagnosis as well as a powerful molecular target for OC treatment.

Implication in OC	References
CS-E displays strong up regulation in primary ovarian carcinomas which is responsible for the poor prognostic parameters, including high tumour grade and advanced FIGO stages.	[115,121]
CS-E can strongly bind to VEGF which is the most important pro- angiogenic stimulator. Furthermore, high CS-E levels correlate to high VEGF, causing further neo-vascularization development in the tumour stroma causing ovarian spheroid formation this spheroid formation is associated with the highly aggressive and invasive characteristics of OC.	[115,116,122]
The elevated CS-E aids the adhesion of tumours as it is responsible for increasing the adhesive properties of adhesion molecules N-cadherin and E- cadherin. In addition, integrins also play a role in adhesion as they can interact directly with CS chains, blocking of integrin receptors result in inhibition of OC cell adhesion.	[115,123]
The overexpression of CS-E improves the adhesive properties of tumour cells.	[115]
CS-E is responsible for the invasion and migration of tumours. MMPs is a group of enzymes responsible for OC progression. The activation and regulation are strongly influenced by CS, Furthermore, CS-E can interact with MPPs such as pro-MMP7, contributing to the activation and metastasis of tumour cells.	[123]
CS-E expression is predominantly seen in the stromal compartment of both primary ovarian carcinomas and metastasis.	[116]
The expression of mRNA for GalNAc4S-6ST, an enzyme which is responsible for the biosynthesis of CS-E, is up-regulated in OC.	[115,124]
CHST15, the only sulfotransferase responsible for biosynthesis of CS-E presents an altered transcription pattern in OC, furthermore increased CHST15 levels lead to increased CS-E levels.	[123,124]
CS has shown to be involved tumour cell proliferation, growth, angiogenesis, adhesion, migration, invasion, and survival of OC tumours.	[123]

Abbreviations in the table are defined as follows: VEGF, Vascular endothelial growth factor; MMPs, Metalloproteinases; GalNAc4S-6ST, Gal-NAc-4-sulphate-6-*O*-sulfotransferase.

**Table 4:** Various antibodies used to target their respective CS isomer.

Antibody	Specificity	Target Selectivity of CS Isomer	References
GD3G7 antibody		- antibody GD3G7 reacted strongly with CS-E, rich in GalNAc4S6S disaccharide units. - minor reactivity with CS-A. - no reactivity was observed with DS, CS-C, CS-D, and HS.	[124]
The mAb 473HD	DSD	- recognizes the characteristic CS structure named the DSD-1 epitope that contains the D-unit.	[124]
mAb CS-56		- reacts with CS-A and CS-C - also reacts preferentially with CS-D - reacts weakly with CS-C, no reactivity with any other CS variants	[124]
mAb MO-225		- reacts strongly with CS-D - moderately with CS-C and CS-E - weakly with CS-A	[130]
473HD	CS-D	- reacted with the hexa- and larger oligosaccharide fractions derived from CS-D	[130]
IO3D9		- reacts with CS-C and weakly with CS-A - strong reactivity with CSE compared to CSA or CSC	[79]
IO3H10		- reacts with CSC - reacts with CSA	[79]
IO3H12		- reacts with CSC - reacts with CSA	[79]
12C and E-18H	CS-E specific	- no reactivity with any other CS isomer	[129]
GD3A11	CS-E specific	- no reactivity is observed with other immobilized GAGs such CS-A, CS-B, CS-C, CS-D, HS, and heparin	[120]

**Table 5:** The relationship of CD44 and HA to the ovarian cancer development.

Implication in OC	References
CD44 is proposed to be involved in increased motility of cancer cells as well as differentiation of cancer stem cells	[91]
Co-overexpression of CD44 and multiple drug resistance proteins such as MDR1 and MDR2 correlate to OC progression.	[138]
RHAMM and CD44 are vital components for tumour progression.	[139]
When HA synthase and HA are down regulated directly using siRNAs it causes impaired cytoskeletal activation and decreased migration of tumours.	[141]
HA mediates the physical linkage between CD44s and Her2/ErbB2 tyrosine kinase, which results in the rapid stimulation of ovarian carcinoma cell growth.	[140]
Elevated CD44 expression is observed in OC in contrast to benign and borderline tumours.	[138]
Elevated HA concentration provides and additional growth advantage of primary ovarian tumours due to the cells ability to produce a HA rich stroma environment.	[142]
The high stromal HA levels are significantly associated with poor differentiation, serous histological type, advanced stage, and large primary residual tumour in epithelial OC.	[133]
CD44 expressions correlate with the expression of the drug efflux pump Pgp causing resistance and progression of metastases.	[91]
CD44 inhibition following treatment of the CD44 monoclonal antibody inhibits OC cell motility no significant impact on invasion	[3]
Anti-CD44 antibody has been shown to decrease the number of total peritoneal OC metastases in mice.	[3]
